# Enhanced Tuberculosis Infection Treatment Outcomes after Implementation of QuantiFERON®-Gold Testing

**DOI:** 10.1371/journal.pone.0138349

**Published:** 2015-09-15

**Authors:** Aldo Crossa, Jason Kessler, Tiffany G. Harris

**Affiliations:** 1 New York City Department of Health and Mental Hygiene, New York, New York, United States of America; 2 Mailman School of Public Health, Columbia University, New York, New York, United States of America; Hopital Raymond Poincare—Universite Versailles St. Quentin, FRANCE

## Abstract

**Background:**

Use of the tuberculin skin test (TST) for diagnosis of latent tuberculosis infection (LTBI) among individuals who received the Bacille Calmette-Guérin (BCG) vaccine is complicated by its potential cross-reaction with TST antigens which may cause false-positive results and lead to patient and physician reluctance to initiate LTBI treatment. QuantiFERON®-TB Gold (QFT-G) lacks this cross-reaction. We sought to study the impact of implementing QFT-G testing in 2006 on LTBI treatment initiation and completion at NYC chest clinics.

**Methods:**

QFT-G results from 10/2006–12/2008 in NYC Department of Health and Mental Hygiene chest clinics were obtained from the electronic medical record system. The proportions of patients who initiated and completed treatment among patients tested with QFT-G were compared to those tested with TST from 10/2004–9/2006.

**Results:**

Among 36,167 patients tested with QFT-G, 2,300 (6%) tested positive, 33,327 (93%) tested negative, and 540 (1%) had an indeterminate result. Among those who had a positive QFT-G test and deemed eligible, 985 (80%) initiated LTBI treatment and 490 (40%) completed treatment. Historically, among patients tested with TST, 7,073 (19%) tested positive (p<0.0001 compared to QFT-G); 3,182 (79%) of those eligible initiated LTBI treatment and 1,210 (30%) completed treatment (p<0.0001 compared to QFT-G).

**Conclusions:**

QFT-G implementation increased the proportion of patients completing LTBI treatment. Additional studies are needed in more settings to determine whether using QFT-G leads to a sustained increase in treatment completion.

## Introduction

Tuberculosis (TB) rates have declined in the United States (US) since the late 1990s as the HIV epidemic waned in addition to improvements in TB control infrastructure [1]. However, TB elimination is far from being achieved, partly due to the large reservoir of individuals with latent TB infection (LTBI). A key component of the US’s TB elimination strategy is the identification and treatment of those at highest risk of developing TB including immunosuppressed persons, recent immigrants to the US with LTBI, and contacts of known TB cases [1–3]. In the US an estimated 4.2% of people are infected with TB [4].

For LTBI treatment to be an effective strategy for achieving TB elimination, a sizeable proportion of those with LTBI must complete a 3–9 month treatment regimen. A large North American multisite study of LTBI screening and treatment found that only 46.6% of those testing TST positive completed treatment [5], a proportion that has remained unchanged during the last decade [6]. Shorter LTBI treatment regimens have been recommended, and may be associated with increased treatment completion [7–10].

Barriers to initiation and completion of LTBI treatment include provider and patient misperceptions about LTBI and TB disease risk, as well as patients with LTBI doubting the accuracy of diagnostic tests such as the TST. In addition, therapy duration and potential drug adverse events may also play a role in poor treatment initiation and adherence rates [11,12]. The medical training and background of healthcare providers can also affect their perception of diagnostic accuracy and disease risk and whether they recommend treatment [13]. Furthermore, directly observed therapy (DOT), which can greatly improve treatment adherence, is not standard for LTBI treatment as it is for active TB.

Due to the specific mixture of antigens used in the TST [14], individuals who have received Bacillus Calmette-Guérin (BCG) vaccination or are infected with non-tuberculosis mycobacteria (NTM) may have a false-positive TST result. Interferon-gamma release assays (IGRAs) overcome some of the TST’s shortcomings by using *Mycobacterium tuberculosis (M*. *tb)* complex-specific antigens that do not cross-react with BCG or with most NTMs.

Improved specificity of IGRAs may result in increased LTBI treatment initiation and completion. In a study conducted among healthcare workers, IGRA testing was associated with increased initiation of LTBI treatment [15]. Use of IGRAs in the public health setting show mixed results. In a recent study of contacts to TB cases, those with a positive IGRA were more likely to both initiate and complete LTBI treatment compared to those who with a positive TST [16]. However, in another study among patients with suspected LTBI, IGRA testing was not associated with treatment initiation or completion [17]. The objective of our study was to evaluate the impact of utilizing QuantiFERON®-TB Gold (QFT-G) on LTBI treatment initiation and completion at NYC Department of Health and Mental Hygiene (DOHMH) chest clinics.

## Methods

### Study subjects and data collection

In October 2006, the NYC DOHMH began implementing QFT-G (Cellestis Limited, Carnegie, Victoria, Australia) as the standard test for *M*. *tb* infection for all patients aged ≥1 year at its chest clinics. At the time, the clinics provided testing for contacts of TB cases, other high-risk individuals, and for those needing testing to satisfy administrative requirements (e.g. employment, school). These clinics also provided evaluation, medical care, and treatment for LTBI and active TB [18]. The study population included patients who were tested with QFT-G at any of the 10 DOHMH chest clinics from October 1, 2006−December 31, 2008. This group was compared to a historical cohort of patients tested with TST at the same clinics from October 1, 2004−September 30, 2006.

QFT-G testing was performed and interpreted as positive, negative, or indeterminate, per the FDA-approved package insert and the US Centers for Disease Control and Prevention (CDC) guidelines [19,20]. TSTs were performed using the Mantoux method. Results were interpreted by trained staff 48–72 hours later as positive or negative based on American Thoracic Society (ATS)/CDC guidelines [21]. Patients with a positive result or those at high risk for developing TB disease (e.g., HIV-infected) were referred for chest radiograph and evaluation by a physician.

LTBI diagnosis was based on the QFT-G or TST result, chest radiograph(s), physical examination, TB risk factor and exposure history, and, in certain circumstances, sputum examinations [21,22]. After ruling out TB disease, the evaluating physician determined whether treatment for LTBI was indicated in accordance with national guidelines and the NYC TB Clinical Policies and Procedures [22–24]. The standard LTBI treatment regimen was 9 months of isoniazid; alternative regimens were used as appropriate [22,25].

All data used in the study were abstracted from the clinic electronic medical record (EMR) system. Available demographic information included birth date, sex, race/ethnicity, country of birth, primary language, time in the US (foreign-born patients only) and reason for testing. We categorized the reason for test as contact/non-contact; this did not affect the overall estimates of test type (not shown).

Patients who had TB disease or who had a documented history of TB disease were excluded from all analyses, as were patients who received both a QFT-G and TST test. For patients with multiple tests of the same type, only the last result (and its associated clinical and demographic characteristics) was used because most of these patients were contacts receiving a window and post-window test and the last test was considered the final result. In addition, patients with a positive test who had a documentation of a chest radiograph and a medical evaluation in the EMR were considered fully evaluated. Patients missing either a chest radiograph or a medical evaluation were considered to be partially evaluated. If neither existed, the patient was categorized as not evaluated.

### Statistical analysis

For each time period (QFT-G and TST), the percentages of individuals who had a positive test, received a complete evaluation following a positive test, had an indication for LTBI treatment as determined by a clinic physician, started on LTBI treatment, and completed LTBI treatment were calculated. Comparisons between the time periods were conducted using Pearson’s chi-square statistic.

The proportion of patients initiating LTBI treatment was calculated as the number of patients with a positive test (QFT-G or TST) who initiated treatment divided by the number with a positive test and an indication for treatment. Bivariate and multivariate Poisson regression models were used to determine whether the percentage of patients that started LTBI treatment differed during the QFT-G and TST periods. Generalized Estimating Equations (GEE) were used to account for similarities between patients tested at the same clinic. Any patient whose final QFT-G result was indeterminate or missing or whose final TST result was not read were excluded from this analysis. Potential confounders evaluated included the following: recent exposure to an active TB case (i.e. being a contact), age at testing, sex, birth in the US, time in the US among foreign-born, primary language, and race/ethnicity. Unadjusted risk ratios (RR) were first estimated for each variable and then adjusted models were constructed with factors with a *p*-value <0.2 as well as year of test to account for any temporal changes in LTBI treatment initiation and completion during the study period including other programs initiated by the NYC DOHMH aimed at increasing LTBI treatment completion.

The proportion completing LTBI treatment was calculated by dividing the number of patients completing treatment by either (a) the number of patients with an indication for treatment or (b) those with indication who initiated treatment. An analysis similar to the one described above was conducted for completion of LTBI treatment. Patients who did not have a documented treatment outcome (TST: 987, QFT-G: 200) were assumed to have not completed treatment.

All analyses were performed using SAS version 9.2 (SAS Institute, Cary, NC, USA).

### Ethics Statement

The New York City Department of Health and Mental Hygiene Institutional Review Board approved the study. No patient consent was obtained for this study as a waiver was received from the aforementioned IRB.

## Results

### LTBI testing and treatment outcomes

**Fig 1:**
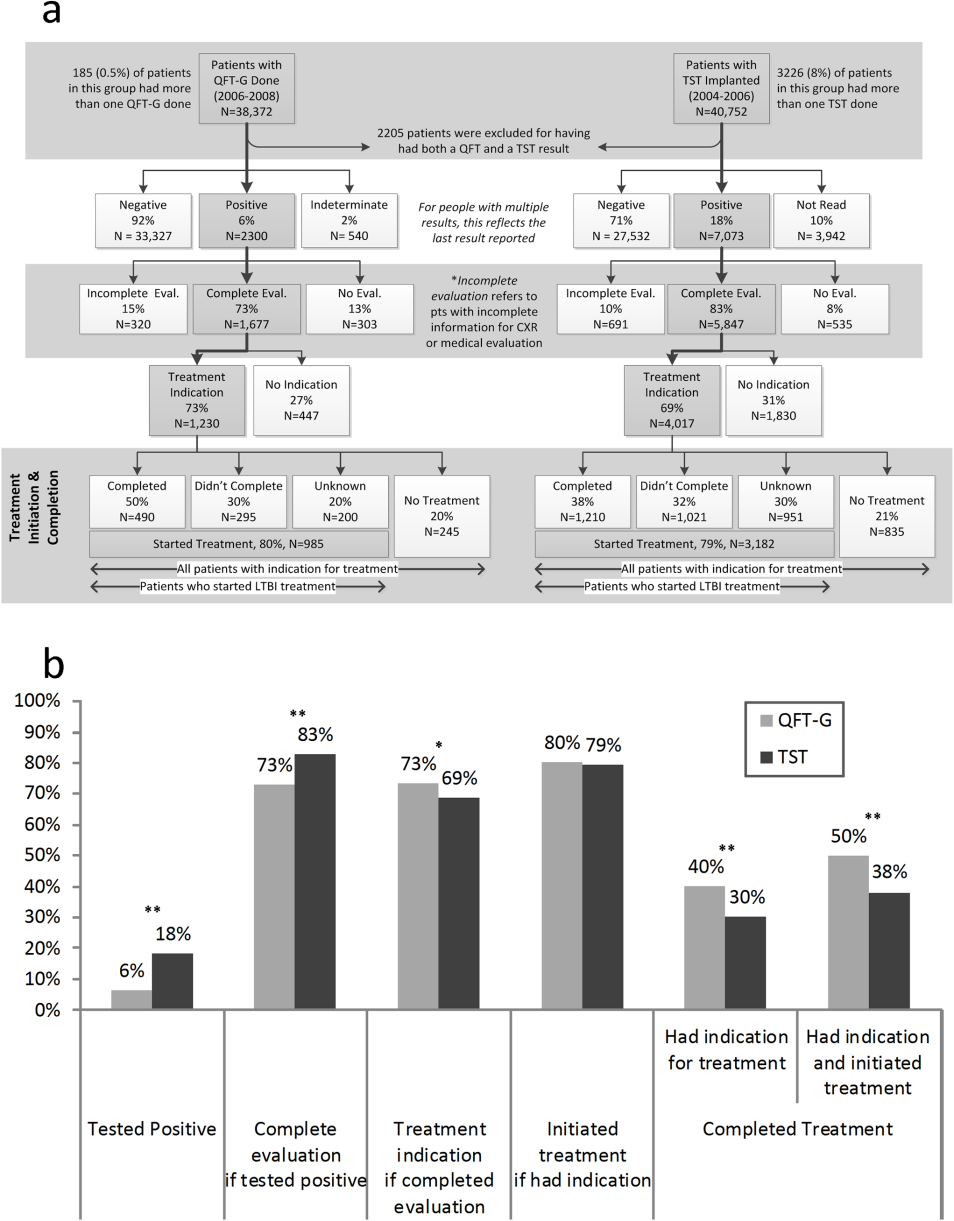
From testing to treating LTBI in New York City Department of Health and Mental Hygiene chest clinics by QFT-G (2006–2008) or TST (2004–2006). **(a)**. Flowchart representing the flow of patients from testing to treatment for LTBI in the utilization periods (QFT-G and TST). **Complete Evaluation**: evaluation by a physician and had a chest radiograph. **Incomplete Evaluation**: patients who were evaluated by a physician or had a chest radiograph but not both. **LTBI Treatment Completion**: 9 months of isoniazid or completion of an alternative regimen, per ATS/CDC and NYC DOHMH guidelines at that time. **(b)** Comparison of final LTBI test results, treatment initiation, and treatment outcomes between the utilization periods (QFT-G and TST). The bars represent (from left to right) the percentage of patients who tested positive for LTBI; of patients who tested positive, the percentage evaluated for LTBI treatment; of those evaluated, the percentage that had an indication for treatment; percentage of patients with treatment indication who initiated treatment for LTBI; percentage that completed treatment for LTBI among those who had an indication, and the percentage that completed treatment among all those with an indication for treatment who started treatment. [*] P<0.001, [**] P<0.0001

From October 1, 2006−December 31, 2008, 38,372 patients were tested with QFT-G at the 10 chest clinics and from October 1, 2004−September 30, 2006, 40,752 patients received 43,978 TST implants (Fig 1A). There were 2,205 patients who received both tests and were excluded (96% tested negative on both QFT-G and TST). Among patients tested only with QFT-G, 0.5% (185/38,372) had multiple tests performed and during the TST period, 8% (3,226/43,978) had >1 TST implanted; for these patients the last result was used. The percentage was substantially higher for TST because it reflects patients not returning in 48–72 hours for the TST reading and needing to be tested again. The baseline patient characteristics by LTBI time period are shown in Table 1. Of the final 36,167 patients (38,372 minus 2,205 receiving both tests) in the QFT-G time period, 33,327 (92%) tested negative, 2,300 (6%) tested positive, and 540 (2%) had an indeterminate result. Of the final 38,547 patients tested with TST, 27,532 (71%) tested negative, 7,073 (18%) tested positive, and 3,942 (10%) did not return for the reading and did not have another TST during the study period (Fig 1A). Overall, the percent that tested positive with QFT-G was significantly lower than that with TST (6% vs. 18%, p<0.0001, Fig 1B).

**Table 1:** Baseline characteristics of persons undergoing testing for LTBI during QFT-G (2006–2008) and TST (2004–2006) utilization in New York City Department of Health and Mental Hygiene chest clinics.^a^

		QFT-G		TST	
		(N = 36,167)		(N = 38,547)	
		N	%	N	%
**Sex**					
	Female	19,511	54	21,493	56
	Male	16,656	46	17,054	44
**Age group (years)**					
	1–4	237	1	482	1
	5–9	1,142	3	682	2
	10–14	2,997	8	1,860	5
	15–19	4,973	14	4,622	12
	20–49	22,973	64	26,600	69
	50–65	3,415	9	3,835	10
	>65	430	1	466	1
**Race/Ethnicity**					
	Non-Hispanic white	4,501	12	4,785	12
	Hispanic (Any)	10,707	30	12,496	32
	Non-Hispanic black	14,705	41	16,329	42
	Asian	4,558	13	3,212	8
	Other^b^	1,696	5	1,725	4
**Country of birth** ^c^					
	US	18,982	52	22,446	58
	Non-US	17,185	48	16,041	42
**Time in US (foreign-born only)** ^c^					
	0–1 years	5,632	33	5,250	33
	1–5 years	3,257	19	2,785	17
	>5 years	8,296	49	8,005	50
**Primary Language**					
	English	23,895	66	28,159	73
	Non-English	12,272	34	10,388	27
**Contact to Active TB Case** ^d^					
	Yes	1,182	3	1,152	3
	No	34,985	97	37,395	97

QFT-G = QuantiFERON-TB Gold, TST = tuberculin skin test

^a^ Excludes 2205 patients who had both TST and QFT-G performed

^b^ Includes Native American, Pacific Islander

^c^ 60 patients were missing a country of birth and 1 foreign-born patient was missing time in the US.

^d^ Contacts were those exposed to a patient with infectious TB disease and were tested as part of a NYC DOHMH contact investigation. Non-contacts included patients tested for any other reason

Among the 2,300 individuals who tested QFT-G positive, 1,677 (73%) had a complete clinical evaluation, and 1,230 (73%) of these individuals had an indication for treatment. Among the 7,073 who tested positive with TST during the historical comparison period, 5,847 (83%) had a full clinical evaluation and 4,017 (69%) of these fully evaluated patients had an indication for treatment. Patients who tested positive with QFT-G were significantly more likely to have an indication for treatment compared to those tested with TST (73% vs. 69%; p = 0.002; Fig 1A).

Among patients who had an indication for LTBI treatment, 80% (985/1,230) in the QFT-G period and 79% (3,182/4,017) initiated treatment in the TST period. This was not statistically significant in the bivariate analysis (*RR* = 1.01, *95% CI* = 0.98–1.10) but became significant after adjusting for other covariates [*adjusted RR (aRR) =* 1.10, *95% CI* = 1.03–1.17]. The reasons and frequencies for not starting treatment despite indication were not statistically different between the TST and QFT-G time periods (overall *P =* 0.243). The most common reasons for not starting treatment included refusal (QFT-G: 53%, TST: 55%); loss to follow-up or moving (QFT-G: 31%, TST: 25%); no reason provided in the EMR (QFT-G: 8%, TST: 9%); or other reasons including transferring to another healthcare provider (QFT-G: 6%, TST: 8%).

Among those with an indication for treatment, patients in the QFT-G period were 32% more likely to complete treatment compared to the TST period (*RR* = 1.32, *95% CI* = 1.16–1.51; Table 2). After adjusting for other covariates, patients from the QFT-G period were 75% more likely to complete treatment (*aRR* = 1.75, *95% CI* = 1.49–2.06). Among those who initiated treatment, those tested with QFT-G were 60% more likely to complete than the TST group (*aRR* = 1.60, *95% CI* = 1.36–1.87). In addition, younger patients as well as foreign-born and non-English speakers were more likely to complete treatment compared to older, US-born and English speaking patients respectively. Contacts were more likely to complete treatment than non-contacts (*aRR* = 1.35, *95% CI* = 1.14–1.52).

**Table 2:** LTBI treatment completion among patients with an indication for LTBI treatment, New York City Department of Health and Mental Hygiene chest clinics during QFT-G (2006–2008) and TST (2004–2006) utilization periods.

		Completed treatment		Did not complete treatment		
		(N = 1,700)		(N = 3,547)		
		N	%	N	%	aRR_Q/T_ ^e^
**Test Type**						
	QFT-G	490	40	740	60	**1.75 (1.49–2.06)**
	TST	1,210	30	2,807	70	Ref
**Sex**						
	Female	853	32	1821	68	—
	Male	847	33	1726	67	—
**Age group (years)**						
	1–4	7	33	14	67	**2.31 (1.44–3.72)**
	5–9	35	50	35	50	**3.19 (2.30–4.44)**
	10–14	170	45	209	55	**2.99 (2.33–3.85)**
	15–19	353	39	555	61	**2.52 (1.93–3.29)**
	20–49	962	30	2,198	70	**2.05 (1.72–2.46)**
	50–65	160	25	469	75	**1.73 (1.37–2.18)**
	>65	13	16	67	84	Ref
**Race/Ethnicity**						
	Non-Hispanic white	64	22	223	78	**Ref**
	Hispanic (Any)	658	35	1,235	65	**1.41 (1.22–1.64)**
	Non-Hispanic black	458	26	1,281	74	**1.22 (0.93–1.59)**
	Asian	443	42	620	58	**1.63 (1.39–1.90)**
	Other^a^	77	29	188	71	**1.29 (1.07–1.57)**
**Country of birth** ^b^						
	US	222	24	711	76	Ref
	Foreign	1477	34	2834	66	**1.18 (1.01–1.38)**
**Time in US (foreign-born only)** ^b^						
	0–1 years	637	38	1020	62	Ref
	1–5 years	371	36	674	65	0.95 (0.87–1.06)
	>5years	469	29	1,140	71	**0.81 (0.74–0.90)**
**Primary Language**						
	English	668	27	1,808	73	Ref
	Non-English	1,032	37	1,739	63	**1.13 (1.05–1.22)**
**Contact to an active TB case**						
	Yes	170	45	212	56	**1.35 (1.14–1.59)**
	No	1,530	31	3,335	69	Ref

QFT-G = QuantiFERON-TB Gold, TST = tuberculin skin test

^a^ Excludes 2205 patients who had both TST and QFT-G performed

^b^ Includes Native American, Pacific Islander

^c^ 60 patients were missing a country of birth and 1 foreign-born patient was missing time in the US.

^d^ Contacts were those exposed to a patient with infectious TB disease and were tested as part of a NYC DOHMH contact investigation. Non-contacts included patients tested for any other reason.

^e^ Estimates of relative risk were adjusted for age at test, reason for test, primary language (English, not English), birth in the US, race/ethnicity and year that the test was performed. Sex was excluded based on the results of the bivariate analysis.

To examine whether the analysis of treatment initiation was affected by excluding those without documentation of having a complete evaluation, we re-ran the analysis including these patients. With the inclusion of these patients, QFT-G testing was not associated with increased initiation (*aRR* = 1.06, *95% CI* = 0.99–1.13). When these patients were included in the analysis of treatment completion those tested with QFT-G remained more likely to complete among patients with indication for treatment (*aRR* = 1.68, *95% CI* = 1.40–2.02) and patients who started treatment (*aRR* = 1.60, *95% CI* = 1.33–1.90).

## Discussion

The utilization of QFT-G in NYC DOHMH clinics was associated with several important changes related to LTBI diagnosis and treatment. First, during the initial 2 years of its use, the number of patients diagnosed with LTBI declined by 68% from 18% when TST was used to 6% with QFT-G. This finding is consistent with other studies of QFT-G and with QFT-G being a more specific test than the TST in populations that have received BCG [15,26–28] and have a low risk of TB infection. Second, the percentage of patients completing LTBI treatment among those with indication increased significantly from 30% to 40% compared to when TST was used. Our finding of a 40–50% completion rate among treatment initiators is consistent with other studies of LTBI treatment with TST and IGRAs, and illustrates the challenges posed by LTBI treatment, including long treatment duration, associated side effects, and treatment of an asymptomatic condition [29–32].

Our findings indicate that using IGRAs for screening purposes in settings with relatively low rates of TB infection could have several advantages over the TST beyond eliminating the need of a return visit for reading. Having fewer patients test positive will result in a reduction in needed resources (including personnel time) for the follow-up, evaluation, and treatment of patients with suspected LTBI. The resources saved from the decrease in positivity could be redirected to focus on ensuring those at highest risk for developing active TB initiate and complete treatment. Furthermore, patients will not be unnecessarily exposed to medications or burdened with the difficulties associated with additional clinic visits. Increasing LTBI treatment completion will result in prevention of future TB cases, making IGRAs a potentially more cost-effective strategy for TB prevention than the TST [33–35].

Among patients with an indication for treatment, we found the proportion that initiated treatment for LTBI was significantly increased during the QFT-G testing period. The sensitivity analysis indicates that this result is marginally significant and may be dependent on exclusion criteria based on the patient’s evaluation. Interestingly, we identified that a lower proportion of persons had completed the necessary follow-up evaluation after testing positive with QFT-G than during the historical control period (73% vs. 83%). This is likely because, when a patient’s TST was read as positive, they received a chest radiograph and medical examination the same day, whereas, with QFT-G patients with a positive test were contacted and asked to return for further evaluation.

The improvement in the proportion of those who completed LTBI treatment after diagnosis with QFT-G compared to TST has been documented in other studies as well. Grinsdale *et al*. found that among TB contacts, both initiation and completion of treatment were improved among those tested using QFT-G compared to TST [16]. Similar to our results, Shah et al. found that there was no association between treatment initiation and test type, but unlike our study they also did not find any impact on treatment completion [17]. This study also included non-contacts, suggesting that there may be differential effects on treatment uptake and adherence depending on patient and/or provider’s assessment of TB disease risk and/or biases associated with testing strategy (i.e. providers may be more apt to “believe” a positive IGRA as compared to TST). Associations between perceived risk (on the part of the patient) and adherence to screening or treatment have been demonstrated for a wide variety of conditions [36–38]. Our current study is unable to provide further insight into the reasons why completion rates may be higher after a positive IGRA test result.

This study has several limitations. This analysis was done in the context of a TB control program; therefore, we were only able to compare LTBI treatment initiation and completion among patients tested with QFT-G to a historical group tested with TST and other programs initiated by the NYC DOHMH aimed at increasing LTBI treatment completion could not be taken directly into account though our model did contain a time term to address this. Furthermore, no information was available as to determine why patients were not fully evaluated or if they were considered to have or not have an indication for LTBI treatment a priori. However, there were no major changes in policy at the NYC Bureau of TB Control chest clinics during the study period that would have substantially affected indication for LTBI treatment or the regimens used. Second, data on BCG vaccination status, HIV status, and other comorbidities and risk factors, which have been demonstrated to be related to LTBI treatment outcomes, was not available for analysis [12]. Third, this study was conducted before utilization of the newly recommended short course LTBI treatment regimen [39] which may have important impacts on both uptake and adherence so that percentages initiating and completing treatment from our study may not reflect those that would have been seen with the shorter regimen though this is likely not to have differed by test type. Fourth, because DOT is not the standard of care for LTBI treatment, we do not have verification that patients actually took their medication. However, since patients needed to return monthly to obtain medication, those documented as completing treatment most likely did so. Finally, we did not have information on patient and healthcare provider beliefs and attitudes about LTBI and its treatment, including why LTBI treatment was refused. Strengths of this analysis include a large, population-based study population, a diverse patient population, and data was obtained from a “real world” setting. While the large sample size may have resulted in an “over-powered” analysis we believe the differences demonstrated in treatment completion reflect clinically meaningful (as well as statistically significant) differences.

In summary, routine use of IGRAs for LTBI testing could have important public health implications including increased provider and patient acceptance of LTBI diagnosis and treatment and considerable cost savings due to their improved operating characteristics. Future work should include prospective studies evaluating patient and provider attitudes, beliefs, and their association with LTBI treatment outcomes and LTBI treatment outcomes associated with newer treatment regimens [39]. In addition, interventions targeted at specific high-risk groups will likely be needed if improved rates of treatment success are to be realized.
